# Escitalopram as a modulator of proopiomelanocortin, kisspeptin, Kiss1R and MCHR1 gene expressions in the male rat brain

**DOI:** 10.1007/s11033-020-05806-8

**Published:** 2020-09-10

**Authors:** Artur Pałasz, Aneta Piwowarczyk-Nowak, Aleksandra Suszka-Świtek, Katarzyna Bogus, Łukasz Filipczyk, Alessandra Della Vecchia, Kinga Mordecka-Chamera, Itiana Castro Menezes, John J. Worthington, Marek Krzystanek, Ryszard Wiaderkiewicz

**Affiliations:** 1 Department of Histology, School of Medical Sciences in Katowice, Medical University of Silesia, ul. Medyków 18, 40-752 Katowice, Poland; 2 Department of Anatomy, School of Medical Sciences in Katowice, Medical University of Silesia, ul. Medyków 18, 40-752 Katowice, Poland; 3 Department of Clinical and Experimental Medicine, Section of Psychiatry, University of Pisa, 67, Via Roma, 56100 Pisa, Italy; 4 Department of Neurosciences and Behaviour, Faculty of Medicine, University of São Paulo, Av. Bandeirantes 3900, Ribeirao Preto, SP 14049-900 Brazil; 5 Division of Biomedical and Life Sciences, Faculty of Health and Medicine, Lancaster University, Lancaster, LA1 4YQ UK; 6 Department and Clinic of Psychiatric Rehabilitation, School of Medicine in Katowice, Medical University of Silesia, ul. Ziolowa 45/47, 40-635 Katowice, Poland

**Keywords:** Escitalopram, Proopiomelanocortin, Kisspeptin, Kiss1R, MCHR1

## Abstract

Neuropeptides are important, multifunctional regulatory factors of the nervous system, being considered as a novel, atypical sites of antidepressants action. It has already been proven that some of them, such as selective serotonin reuptake inhibitors (SSRI), are able to affect peptidergic pathways in various brain regions. Despite these reports, there is so far no reports regarding the effect of treatment with SSRIs on brain proopiomelanocortin (POMC), kisspeptin, Kiss1R and MCHR1 gene expression. In the current study we examined POMC, kisspeptin, Kiss1R and MCHR1 mRNA expression in the selected brain structures (hypothalamus, hippocampus, amygdala, striatum, cerebellum and brainstem) of rats chronically treated with a 10 mg/kg dose of escitalopram using quantitative Real-Time PCR. Long-term treatment with escitalopram led to the upregulation of MCHR1 expression in the rat amygdala. Kisspeptin mRNA level was also increased in the amygdala, but Kiss1R mRNA expressions were elevated in the hippocampus, hypothalamus and cerebellum. POMC mRNA expressions were in turn decreased in the hippocampus, amygdala, cerebellum and brainstem. These results may support the hypothesis that these neuropeptides may be involved in the site-dependent actions of SSRI antidepressants. This is the first report of the effects of escitalopram on POMC, kisspeptin, Kiss1R and MCHR1 in animal brain. Our findings shed a new light on the pharmacology of SSRIs and may contribute to a better understanding of the alternative, neuropeptide-dependent modes of antidepressant action.

## Introduction

The potential involvement of proopiomelanocortin (POMC), kisspeptin and MCH signalling in several brain functions, including the affective responses, anxiety mechanisms, memory consolidation, sleep generation and control of autonomic homeostasis has come into consideration. Nevertheless, neuropharmacology of these important, multifunctional neuropeptides is so far poorly investigated.

Proopiomelanocortin (POMC) neurons are involved in the control of eating behaviour and several stress related responses [13]. The expression of the POMC seemed to be initially restricted to hypothalamic nuclei, however low POMC mRNA levels were also detected in the limbic structures including the amygdala and hippocampus that suggest its unknown function in the brain affective mechanisms.

Kisspeptin a C-terminally amidated neuropeptide and ligand of metabotropic Kiss1R (GPR54) receptor play a crucial role in the control of ovarian cycle. The Kiss-1 gene expression is strictly controlled by circulating estrogens and testosterone [11]. Majority of studies were focused on the hypothalamic kisspeptin activity, relatively little is known about potential role of this neuropeptide in other brain regions e.g.in the hippocampus and amygdala where kisspeptin signalling was also recently identified [8].

Melanin-concentrating hormone (MCH) is a cyclic neuropeptide that binds to G-coupled MCHR1 receptor. MCH-expressing perikarya are located mainly in the lateral hypothalamus and zona incerta but their peptidergic axons go to numerous structures such as hippocampus and amygdala [6]. The highest densities of MCHR1 expression were found in the nucleus accumbens (NAc), hippocampus, locus coeruleus, basolateral amygdala, and neocortex, suggesting an involvement of MCH signalling in the various brain functions [3].

Clinically used antidepressants are noted by a large spectrum of their pharmacological effects, that are related to their diverse receptor-dependent mode of action in the various brain structures. Accumulating, but still very limited amount of data suggests, that antidepressants may directly or/and indirectly affect the peptidergic signaling circuits within the various neuronal assemblies.

Escitalopram is an S-enantiomer of citalopram, a selective serotonin reuptake inhibitor (SSRI) with a beneficial pharmacological properties and a satisfactory tolerance profile. Escitalopram has a minimal affinity to serotonin, dopamine, and cholinergic receptors, which highly reduce the range of its potential side effects. SSRI-related changes in energy homeostasis and weight gain are often clinically observed [12, 16], however little is known about peptidergic neuronal pathways which could be additional targets for these medications. There is also no findings describing the potential relationships between POMC, kisspeptin, Kiss1R and MCHR1 expressions and SSRI administration in animal models.

The present experimental paradigm aims to shed light on this area by determining if and how long-term treatment with escitalopram influences the expression of POMC, kisspeptin, Kiss1R and MCHR1 genes in the rat brain. Multifunctional POMC, MCH and kisspeptin hypothalamic neurons are regulated by a common, GnIH-dependent signalling pathways [19]. Numerous functional relationships between these cells make them involved in the large spectrum of central neurochemical processes in the brain. Given all aforementioned features we decided to examine the POMC, kisspeptin, Kiss1-R and MCHR1 mRNA levels jointly.

## Materials and methods

### Animals

The studies were carried out on adult (2–3 months old, 185–220 g) male Sprague–Dawley rats housed at 22 °C with a regular 12/12 light–dark cycle with access to standard Murigran chow and water ad libitum. All procedures were approved by the Local Bioethics Committee at the Medical University of Silesia (decision no. 36/2012) and were conducted in a manner consistent with NIH Guidelines for Care and Use of Laboratory Animals.

### Drug administration and tissue collection

Two groups of rats (n = 4) received intraperitoneal injections with respectively physiological saline and escitalopram at dose 10 mg/kg/day every day for 4 weeks (28 injections). The dose was established according to the articles dealing with the effect of escitalopram on the rat brain neuropeptide signaling [4, 5, 13]. Neuroleptics are administered orally in standard neuropsychiatric practice. However, a distinct majority of preclinical studies were based on intraperitoneal injections to maintain the stable blood concentration of the medication and to avoid its initial cytochrome-P450-dependent biotransformation within the intestinal mucosa. 24 h after the last drug administration, animals were anaesthetized with isoflurane and quickly sacrificed. Samples of hypothalamus, hippocampus, amygdala, striatum, cerebellum and brainstem were microsurgically excised from the brain tissue for RNA isolation.

### Real time-PCR reaction

Total mRNA was extracted from the collected brain tissues via homogenization with an ultrasound homogenizer (Heildoph DIAX 900, Germany) in 1 ml of TRIzol® Reagent (Life Technologies). mRNA isolation was performed using chlorophorm/isopropanol and 75% ethanol with samples finally dissolved in 50 μl of RNAse-free water. Collected mRNA samples were transcribed into cDNA during incubation in buffered solution of reverse transcriptase MMLV-RT with RNAsin, oligo-dT and mix of nucleotides at 42 °C for 60 min. using a DNA Thermal Cycler 480 (Perkin Elmer Inc., Waltham, MA). Initial mRNA solutions contained 5 µg of RNA per 100 µl.

Quantitative Real-Time PCR reaction (qPCR) was performed by FastStart SYBR Green Master (Roche) in a Light Cycler ® 96 (Roche) thermal cycler for 40 rounds. Beta-2-microglobulin (B_2_m) was chosen as a standard internal reference gene. Primer sequences; B_2_m: Forward: 5′–CGAGACCGATGTATATGCTTGC–3′, Reverse: 5′–GTCCAGATGATTCAGAGCTCCA-3′. cDNA was amplified using the TaqMan Gene Expression Assay Kisspeptin (Rn00710914_m1, Applied Biosystems), Kiss1R (Rn00576940_m1, ThermoFisher Scientific) and TaqMan Gene Expression Master mix (4369016, Applied Biosystems). For MCHR assay the following primers were used: F: 5′-TTGCTGTGGTGAAGAAGTCCAAGC, R: 5′–AGTGCCAGACGCCGTTCCCCATGA, for POMC: Forward: 5′–CCAAGCGC TCCACGAGACTT, Reverse: 5′–TTGGGAGCAGGTACCCTC (Sigma-Aldrich). Optimal hybridization temperature was established according to a gradient PCR and was 50 and 59 °C.

### Statistics

Statistical analyses were performed using Statistica (Systat Software). Data are presented as mean ± SEM. Mean differences between experimental groups were analyzed using one-way ANOVA followed by Tukey’s *post-hoc* test. Differences were considered statistically significant at *p* ≤ 0.01 and *p* ≤ 0.05.

## Results and discussion

There was previously suggested that SSRI treatment may affect peptidergic signalling by a modulation of the corticotropin-releasing factor (CRF) pathway and hypothalamic-pituitary adrenal (HPA) axis [5, 10]. Escitalopram administration also increased the concentration of TRH-like peptides in the rat NAc, striatum, cerebellum and brainstem, while TRH concentration was decreased in the NAc [17]. Interestingly, chronic citalopram treatment increased the number of RF-amide related peptide (RFRP) expressing neurons in the rat dorsomedial hypothalamus (DMH) which suggests this SSRI may inhibit the sexual by a modulation of RFRP signalling through serotonin receptors in the DMH [18].

**Fig. 1:**
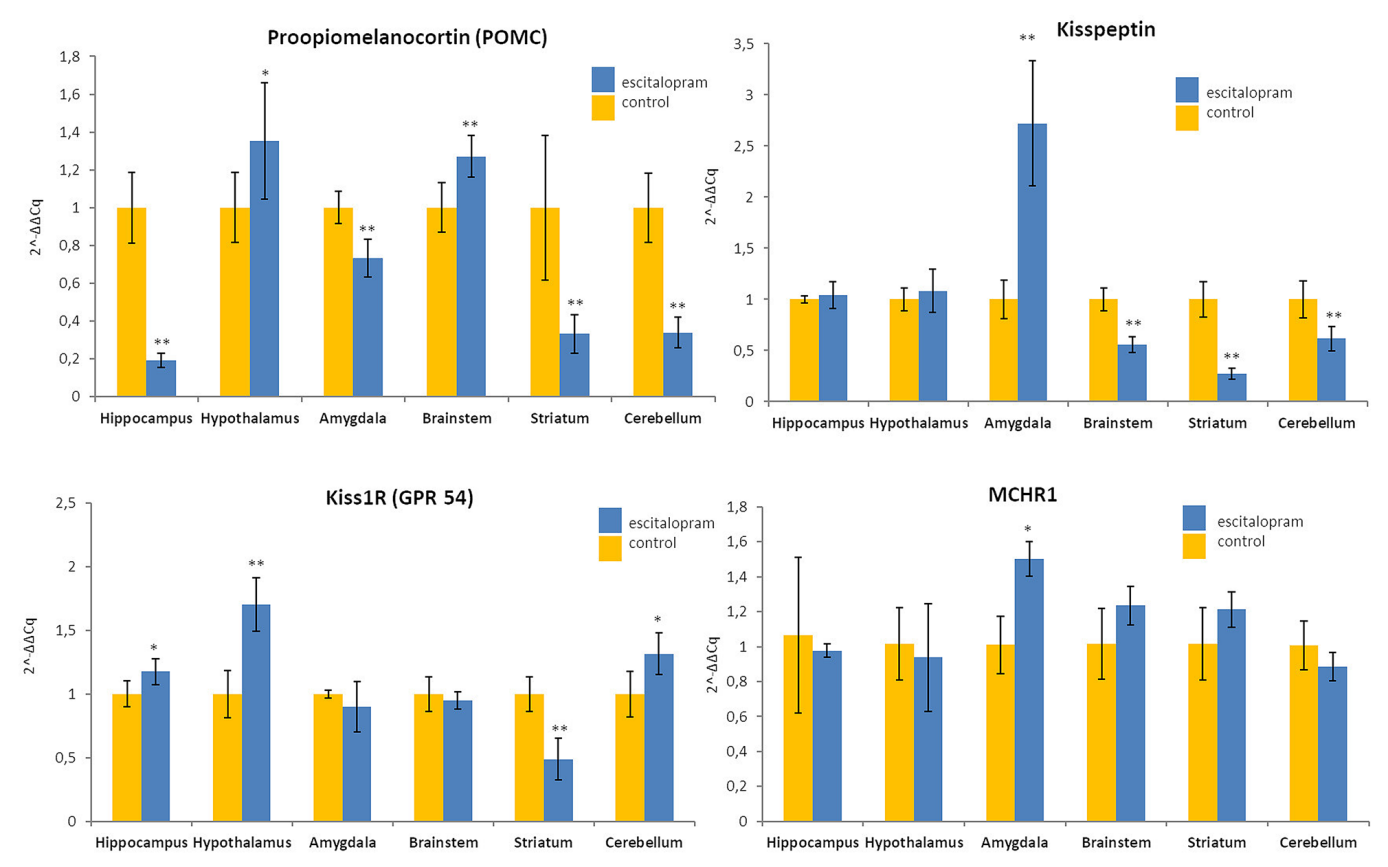
Quantitative PCR results of relative POMC, kisspeptin, Kiss1R and MCHR1 mRNA expression levels in the rat brain. Results were normalized to beta-2-microglobulin reference gene. Data are presented as multiples/decimals of control (1) ± SEM. Differences were considered statistically significant at p ≤ 0.01 (double asterisks) and p ≤ 0.05 (asterisk)

Here we shown that rats treated chronically with escitalopram manifested elevated POMC mRNA expression in the hypothalamus and brainstem (135% and 127% of control respectively), it was mildly decreased in the amygdala (73.4% of control), but highly decreased in the hippocampus, striatum and cerebellum (19,3%, 33,3% and 33,8% of control respectively, Fig. 1). Recent studies reported that fluoxetine increases the ratio between excitatory and inhibitory synaptic contacts on POMC neurons. This effects was parallel to elevated activity of the hypothalamic mTOR pathway. Importantly, administration of rapamycin, an mTOR blocker abolished the effects of fluoxetine on body weight and plasticity of POMC cells, suggesting that SSRI is able to functionally remodel these neurons, via mTOR signaling pathway [2]. There is still no data available about SSRIs effect on POMC expression in the rat brain. An experiment with long-term olanzapine (atypical antipsychotic medication) shows increased POMC mRNA levels in the male rat amygdala [13, 14]. On the other hand, long-term treatment with risperidone decreased the POMC mRNA levels in the rat hypothalamus [7].

Interestingly, rats treated chronically with escitalopram manifested highly elevated kisspeptin mRNA expression in the amygdala (272% of control) but decreased in the striatum, brainstem and cerebellum (27,2%,55,8% and 61,5% of control respectively). The level of Kiss1R mRNA was distinctly increased in the hypothalamus (170% of control), mildly increased in the hippocampus (117%) and cerebellum (131%) but highly decreased in the striatum (48,8%, Fig. 1). Potential effects of antidepressants on the kisspeptin signalling is so far unveiled and there are no articles available dealing with the relations between SSRIs and kisspeptin/Kiss1R expressions in the brain. Our data are only approximate to the study for kisspeptin after chronically administered classical neuroleptics haloperidol and chlorpromazine, where a decrease Kiss-1 expression was noted in the amygdala [15]. Kisspeptin facilitates hippocampal synaptic transmission by activation of MAP kinase (MAPK) pathway in the granular cells of dentate gyrus. It should be especially emphasized, that Kiss-1 gene expression in the hippocampus is considered significantly dependent on the blood estrogene levels [1].

Long-term treatment with escitalopram increased MCHR1 mRNA level in the rat amygdala (150% of control, Fig. 1). This is the first report dealing with SSRI effect on MCHR1 expression in the brain and currently there are no any comparative results available. Interestingly, targeted infusion of MCH into the rat hippocampus and amygdala caused anxiolytic effects and abolished anxiogenic action of L-NOARG, a selective nitric oxide synthase (nNOS) inhibitor [9]. On the other hand chronic stress induced depressive symptoms in mice were related to increased MCH and orexin expression in the basolateral amygdala. Moreover, stereotactic infusion of MCH supressing siRNA into this region inhibited stress-induced depression-like behaviour. Importantly MCHR1 expressing neurons in the basolateral amygdala are functionally connected with central amygdala, NAc and hypothalamic paraventricular nucleus [6].

Present study focuses on analysis of multifunctional neuropeptide gene expressions and provides original data and hypothesize concerning activity of alternative mechanisms, using antidepressants in rat brain. We have to point out several limitations of the study, which has to be taken into account. Firstly, the protein concentrations were not measured and there was relatively small group of rats examined. Behavioural tests were not yet performed but they will be provided in our forthcoming research project. Little is known about extrahypothalamic kisspeptin functions in males in the pharmacological and neuropsychiatric context, a large comparative analysis of the antidepressant effects on the rat brain peptidergic profile should be therefore urgently provided. In this short communication we focused on biochemical/molecular changes only, our conclusions are therefore rather cautious.

**Fig. 2:**
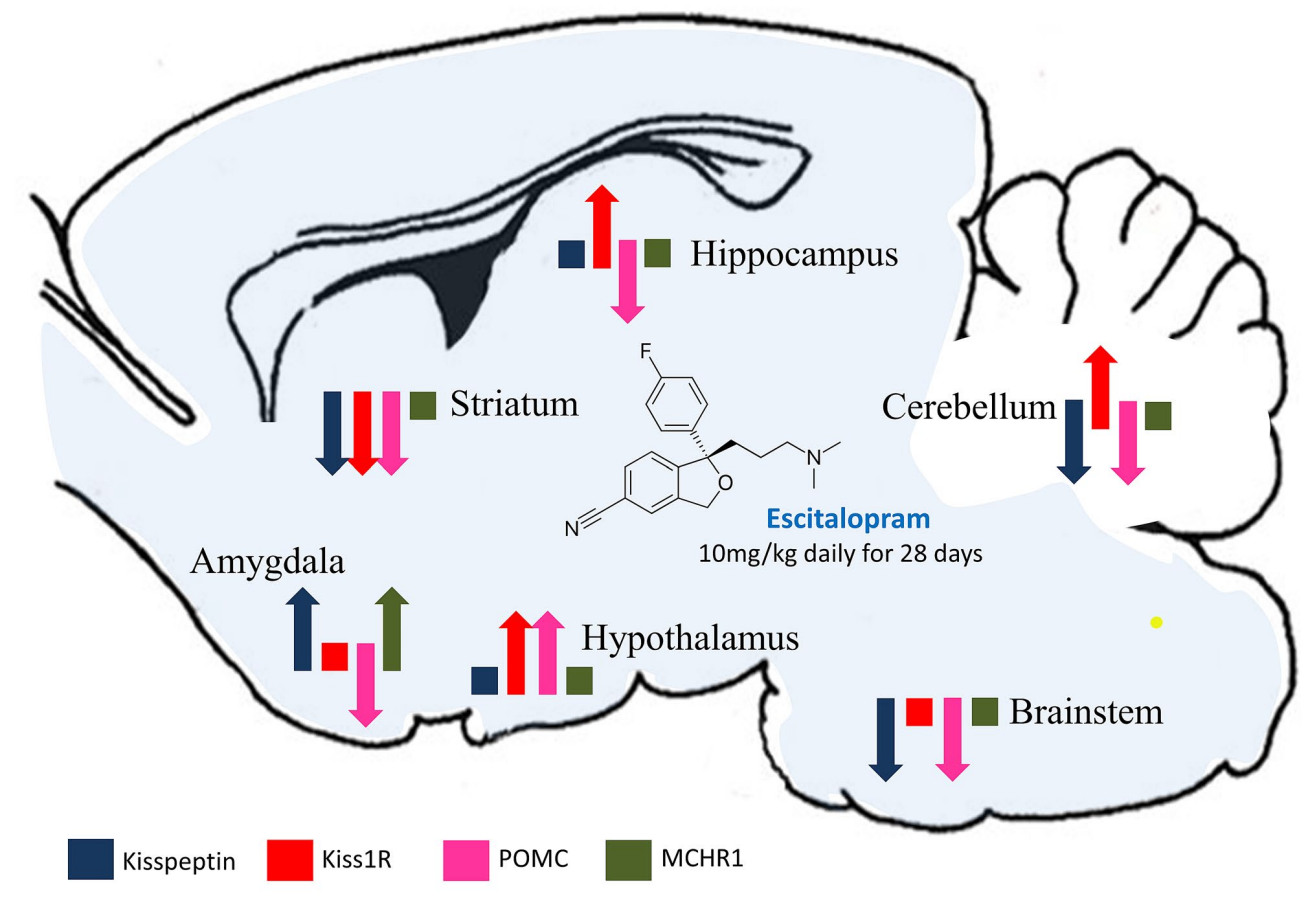
A comparative effect of escitalopram on the POMC, kisspeptin, Kiss1R and MCHR1 mRNAs expression in the main rat brain regions. Changes statistically not significant are presented as squares, significant as arrows

Whether neuropeptide gene expressions are directly related to escitalopram actions or is a secondary effect, has to be definitely investigated in the future. However, in the present study we have shown for the first time changes in the mRNA levels of a POMC, kisspeptin, Kiss1R and MCHR1 mRNA in the main rat brain structures after SSRI drug administration (Fig. 2). The results cautiously indicate that extended escitalopram administration may significantly modulate the regulatory activity of aforementioned neuropeptides in the brain. This may preliminarily suggest an existence of unknown physiological relations connections between these regulatory factors and antidepressant action in animal model, which may contribute to a better understanding of the alternative ways of antidepressant action.
